# Dye-based mito-thermometry and its application in thermogenesis of brown adipocytes

**DOI:** 10.1007/s41048-017-0039-6

**Published:** 2017-05-13

**Authors:** Tao-Rong Xie, Chun-Feng Liu, Jian-Sheng Kang

**Affiliations:** CAS Key Laboratory of Nutrition and Metabolism, Institute for Nutritional Sciences, Shanghai Institutes for Biological Sciences, Graduate School of the Chinese Academy of Sciences, Chinese Academy of Sciences, Shanghai, 200031 China

**Keywords:** Mitochondrial thermometry, Nanothermometry, Thermogenesis, Brown adipocytes

## Abstract

Mitochondrion is the main intracellular site for thermogenesis and attractive energy expenditure targeting for obesity therapy. Here, we develop a method of mitochondrial thermometry based on Rhodamine B methyl ester, which equilibrates as a thermosensitive mixture of nonfluorescent and fluorescent resonance forms. Using this approach, we are able to demonstrate that the efficacy of norepinephrine-induced thermogenesis is low, and measure the maximum transient rate of temperature increase in brown adipocytes.

**Electronic Supplementary Material:**

The online version of this article (doi:10.1007/s41048-017-0039-6) contains supplementary material, which is available to authorized users.

## Introduction

Temperature probing for live cells is challenging, and a lot of efforts have been made to develop nanothermometry to monitor temperatures of living cells (Ye *et al*. 2011; Jaque and Vetrone 2012; Li and Liu 2012; Kiyonaka *et al*. 2013; Kucsko *et al*. 2013; Arai *et al*. 2014, 2015; Homma *et al*. 2015). Recently, all such works have been challenged and criticized by Baffou *et al.*, who have claimed no detectable temperature heterogeneities in living cells (Baffou *et al*. 2014). Apparently, Baffou *et al.* have neglected well-known facts in biology, such as the thermogenesis of brown adipocytes (BA) and mitochondrial role in thermogenesis (Cannon and Nedergaard 2004).

Sympathetic neurotransmitter norepinephrine (NE) can mobilize free fatty acids stored in lipid droplets of BA, and dissipate electrochemical potential energy stored in mitochondrial proton gradient to product heat (Cannon and Nedergaard 2004; Fedorenko *et al*. 2012). Not only as the energy factory of the cells, mitochondrion is the main intracellular site for thermogenesis, which has been targeted for therapy to reduce obesity (Lowell and Spiegelman 2000; Tseng *et al*. 2010). Here, we demonstrate a method of mitochondrial thermometry (mito-thermometry) based on the thermosensitive characteristics of Rhodamine B methyl ester (RhB-ME). With this mito-thermometry, we revealed the low efficacy of NE-induced thermogenesis and the maximum transient rate of temperature increase in BA, and indicated the improper critique of Baffou both practically and theoretically.

## Results

### Evaluation of RhB-ME-based mito-thermometry in HeLa cells

**Fig. 1:**
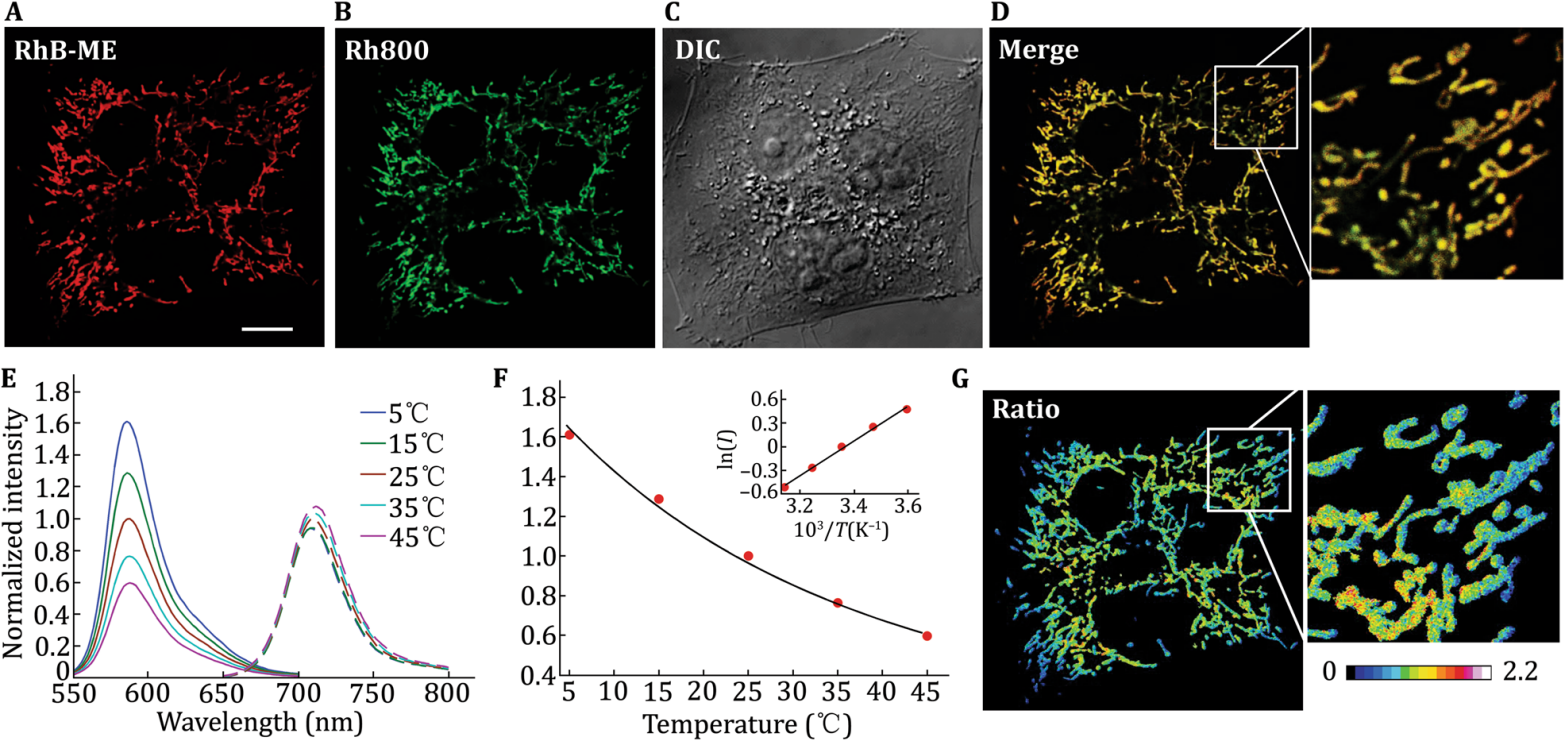
RhB-ME based mito-thermometry in living cells. **A–D** Confocal images of RhB-ME channel (**A**), Rh800 channel (**B**), differential interference contrast (DIC) image (**C**) and merged image (**D**) of stained HeLa cells. *Scale bar*, 10 μm. **E** Emission spectra of 10 μmol/L RhB-ME (*solid lines*) and 10 μmol/L Rh800 (*dashed lines*) from 5 to 45 °C, respectively. **F** Arrhenius fitting for peak values (*red dots*) of RhB-ME spectra. The *black line* indicates a fitting with Arrhenius equation. The *inset* shows the Arrhenius plot. **G** Mitochondrial thermal map of HeLa cells represented by the pseudocolor image of intensity ratio of Rh800 to RhB-ME

Our technique for mito-thermometry employs the thermosensitive and mitochondrial targeting properties of RhB-ME (Fig. 1, synthesis method available online). RhB-ME (Fig. 1A and Supplementary Fig. S1A), a cationic dye like Rhodamine 800 (Rh800, a mitochondrial marker, Fig. 1B and Supplementary Fig. S1B) and many others (Sakanoue *et al*. 1997; Johnson *et al*. 1981), can redistribute within subcellular compartments in response to the negative electric potential, especially in mitochondria (Fig. 1A–D). Mitochondria with large negative membrane potentials lead to spontaneous accumulation of thermosensitive RhB-ME and thermoneutral Rh800 (Fig. 1E) in their matrixes, until reaching equilibrium in accordance with the Nernst distribution law (Sakanoue *et al*. 1997).

The thermochromic transformation of RhB-ME in aqueous solution results in a simple temperature profile, which can be fitted with Arrhenius equation, a single exponential model for the temperature dependence of reaction rates (Fig. 1F). The Arrhenius plot indicates that activation energy of RhB-ME thermochromic transformation is about −4.4 kcal/mol. In living cells, to cancel out the influence of mitochondrial membrane potentials on RhB-ME concentration, the fluorescent intensity ratio of Rh800 to RhB-ME is used to represent mitochondrial thermal profile (Fig. 1G). Both RhB-ME and Rh800 are insensitive to pH, Ca^2+^ or Mg^2+^ (Supplementary Fig. S2). This RhB-ME-based mito-thermometry enables us to acquire the mitochondrial thermal map of HeLa cells at room temperature (RT). The image in Fig. 1G shows mitochondrial temperature gradients in HeLa cells with higher temperature at the center, which can be explained by the geometry of the cells (Fig. 1C).

### The mechanism of RhB-ME thermochromic transformation

**Fig. 2:**
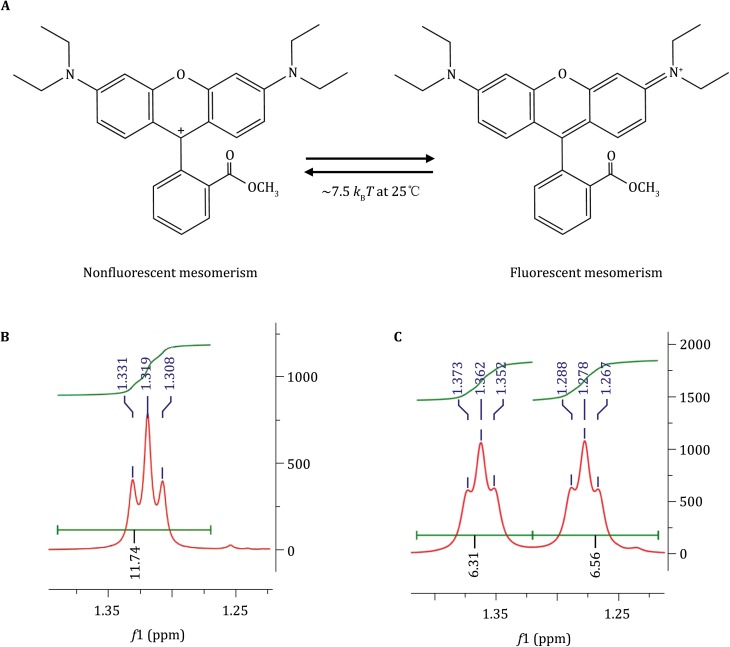
The mechanism of RhB-ME thermochromic transformation. **A** RhB-ME exits as an equilibrium mixture of nonfluorescent and fluorescent resonance forms. **B** and **C** The ^1^H-NMR spectra of four methyl moieties in two diethylamino groups at RT (**B**) and −40 °C (**C**). **B** and **C** show the chemical shifts of 12 protons in the symmetric nonfluorescent form and the asymmetrical fluorescent form, respectively

RhB-ME is a methyl ester derivative of RhB. RhB is cell membrane impermeable (Johnson *et al*. 1981), also thermosensitive (Supplementary Fig. S3), and exists as a mixture of nonfluorescent lactone and fluorescent zwitterion (Hinckley and Seybold 1988). Unlike RhB, our data suggest that RhB-ME exists as an equilibrium mixture of nonfluorescent mesomerism and fluorescent resonance form (Fig. 2A–C), and thermal energy (~7.5 *k*_B_ *T* at 25 °C) can convert single RhB-ME fluorescent molecule to its nonfluorescent form (Fig. 2A). The two diethylamino groups are symmetric in the nonfluorescent form, but asymmetric in the fluorescent form (Fig. 2A). Proton nuclear magnetic resonance (^1^H-NMR) can distinguish between the symmetric form and the asymmetric form by the ^1^H-NMR spectral difference of the 12 protons from four methyl moieties in two diethylamino groups (Fig. 2B–C). The symmetric nonfluorescent form is dominant at higher temperature, which is evidenced by the undistinguishable chemical shifts of 12 protons showing single triplet in the ^1^H-NMR spectra at RT (Fig. 2B and Supplementary Fig. S4). Since decreasing temperature increases the fluorescent intensity of RhB-ME (Fig. 1E, F), the asymmetric fluorescent form of RhB-ME should be dominant at low temperature, and this idea is confirmed by the chemical shifts of 12 protons splitting into double triplets in the spectral data of ^1^H-NMR at −40 °C (Fig. 2C and Supplementary Fig. S5). Compared to thermoneutral Rhodamine 110 (Rh110, Supplementary Fig. S6) and Rh800 (Supplementary Fig. S1B), RhB (Supplementary Fig. S3) or RhB-ME (Supplementary Fig. S1A) has extra four ethyl moieties for two amino groups, which may free for torsional motion (Karstens and Kobs 1980). Thus, our findings demonstrate that thermochromic activation energy of RhB and RhB-ME is contributed to the torsional motion of diethylamino groups, which stabilizes the nonfluorescent form.

### Study the thermogenesis of BA with RhB-ME-based mito-thermometry

To evaluate and make use of RhB-ME based mito-thermometry, we applied it to study the thermogenesis of BA. For thermogenic studies of BAT or BA, calorimeter and oxygen consumption rate (OCR) have been frequently used to evaluate heat production (Clark *et al*. 1986; Wikstrom *et al*. 2014), but both are indirect and might be cumbersome (Cannon and Nedergaard 2004). Although the genetically coded thermometry is versatile for organelle targeting, due to low transfection efficiency of BA, it is difficult to be used for collecting large-scale datasets, which are usually necessary for experiments with large variations. Since there is no need for transfection, injection, or elaborate equipment, dyes (Arai *et al*. 2015; Homma *et al*. 2015) and RhB-ME-based mito-thermometry demonstrated in this study make it easier for large-scale data acquisition, and are capable of detecting thermogenic responses at mitochondrial level.

**Fig. 3:**
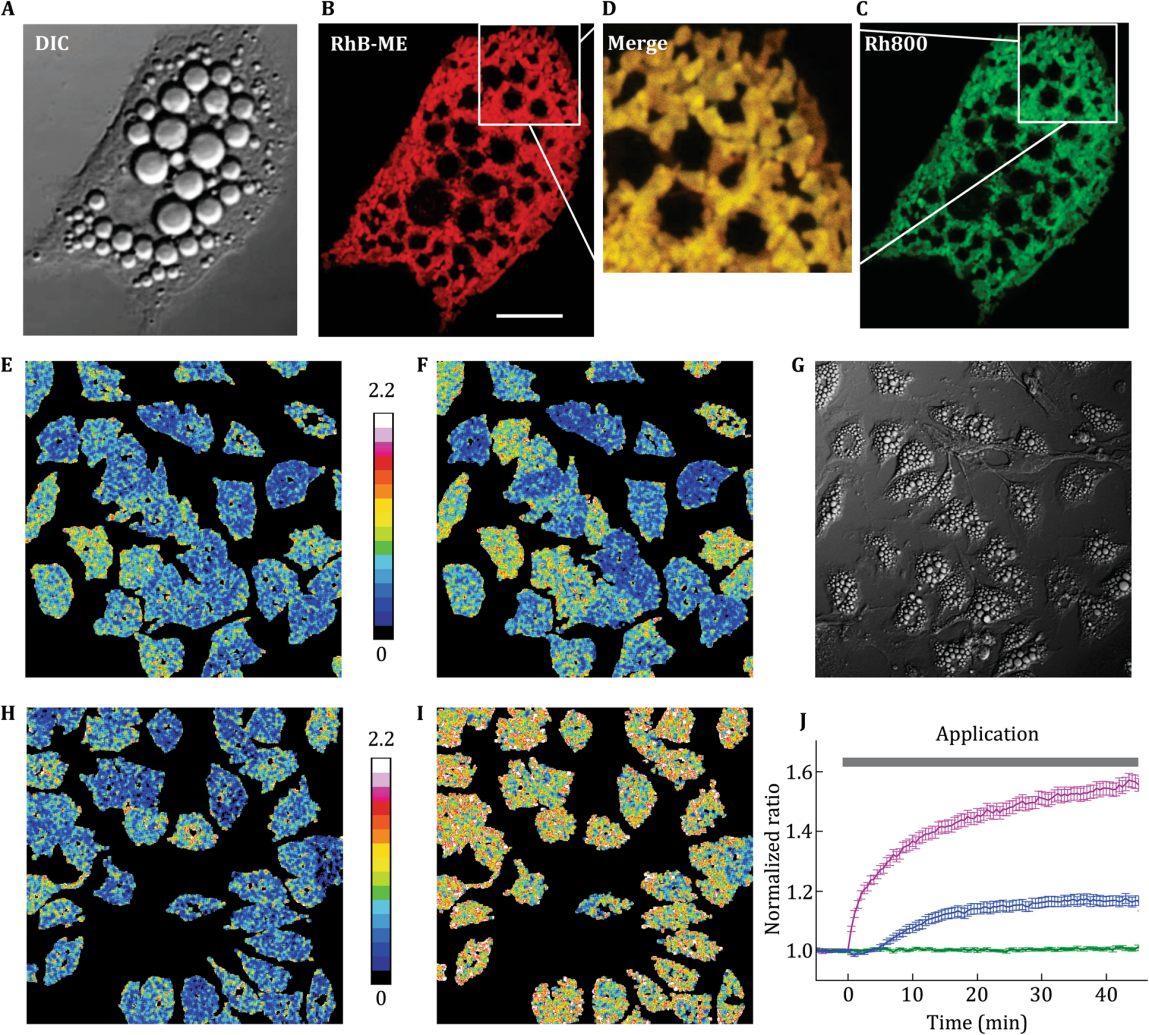
Mito-thermometry reveals low efficacy of NE-induced thermogenesis in BA. **A–C** Confocal images of DIC image (**A**), RhB-ME (**B**), Rh800 (**C**) in stained BA. *Scale bar*, 10 μm. **D** Zoomed and merged image shows crowded mitochondria in BA. **E** and **F** Representative thermal ratio images are shown for the moments before and after NE treatment respectively. *Scale bar*, 20 μm. **G** DIC image of BA. **H** and **I** Representative thermal ratio images are shown for the moments before and after CCCP treatment, respectively. **J** NE (0.1 μmol/L, *blue line*, *n* = 118 cells in four experiments) and CCCP (10 μmol/L, *magenta line*, *n* = 88 cells in three experiments) induced thermogenesis in BA. The *green line* shows the control results of the solvent treatment in BA (*n* = 93 cells in three experiments). All data points in figures represent mean ± S.E.M

As illustrated in Fig. 3A–D, RhB-ME, like mitochondrial marker Rh800, is accumulated in numerous mitochondria of primary cultured BA. The thermogenic responses of BA, represented by the fluorescent intensity ratio of Rh800 to RhB-ME, are evoked by 0.1 μmol/L NE in minutes (Fig. 3E–G, Supplementary Video S1 and S2). Compared to 10 μmol/L carbonyl cyanide m-chlorophenylhydrazone (CCCP, a proton uncoupling agent, Fig. 3H, I and Supplementary Video S3), 0.1 μmol/L NE shows markedly lower thermogenic efficacy (Fig. 3J). In addition, 0.1 μmol/L NE-induced thermogenesis in BA shows large cell-to-cell variation (Fig. 3E, F, Supplementary Video S1 and S2). Only 59.4 ± 15.9% (69 of 118, mean ± S.D.) of NE treated BA show thermogenic responses, which accounts for the low efficacy of 0.1 μmol/L NE-induced thermogenesis in BA (Fig. 3E, F).

## Discussion

Baffou *et al.* have criticized all methods for temperature imaging in living cells (Baffou *et al*. 2014), which have based on a conclusion “temperature increase should be on the order of Δ*T* ~ 10^−5^ K” governed by the heat diffusion equation at one-dimensional steady-state conditions for cell. However, “Δ*T*” is a spatial temperature gradient independent of time in Baffou’s model rather than “temperature increase” (over time), so it is inappropriate to apply a spatial gradient “Δ*T*” to discuss thermogenesis in living cells, a temporal process in time-variant systems. In addition, such small spatial temperature gradient (10^−5^ K) would result in negligible heat flows within cell.

Actually, heat sources in cell would be able to increase their temperature at a transient rate, or at a lasting rate (under a condition with negligible heat dissipation) in the order of ~10^−2^–10^−1^ K/s, such as the rate (Δ*T*/Δ*t*) of mitochondrial temperature change (Δ*T*), can be calculated with the Eq. 1:

1 $$\Delta T/\Delta t = P/C_{\text{P,v}}v,$$

which is deriving from the definition of heat capacity, and where *C*_P,v_ is the volumetric heat capacity of water (4.1796 J/(cm^3^K)); *v* is a mitochondrial volume of 1 μm^3^; the average heat power (*P*) for a cell is 100 pW, which can reach 1 nW for brown fat cells (Baffou *et al*. 2014), and then the *P* of single mitochondrion is taken as 0.1–1 pW assuming ~1000 mitochondria per cell.

**Fig. 4:**
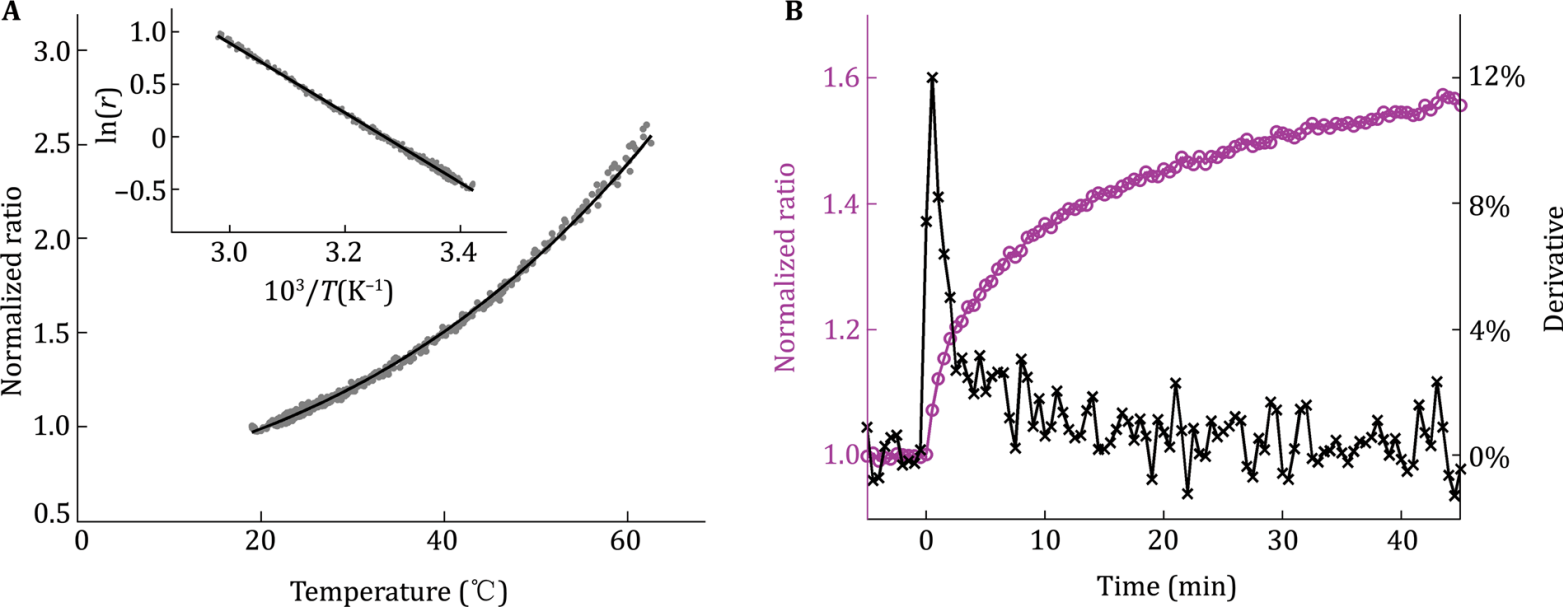
Maximum transient rate of mitochondrial temperature increase in BA induced by 10 μmol/L CCCP. **A** Ratiometric confocal imaging data of 10 μmol/L RhB-ME and 10 μmol/L Rh800 mixed in aqueous solution (from 18.9 to 62.9 °C). The *scatter plot* of fluorescent ratios (*gray dots*) of Rh800 to RhB-ME versus temperature monitored with thermocouple (Pt100A). The *black solid line* is a fit with Arrhenius equation. The *inset* shows the Arrhenius plot. **B** Maximum transient rate of BA-mitochondrial temperature increase induced by 10 μmol/L CCCP. The ratio (*magenta line*) is the same raw dataset in Fig. 3G. The derivative (*black line*) of the ratio data is calculated by numerical differentiation, and the peak value is about 12%/min. Since all ratio data are normalized with the data acquired at 33 °C (306.15 K), the change rate of normalized ratio at this temperature has been theoretically (see details in **Method**) and experimentally determined as 3.5%/K (**A**); thus, the maximum transient rate of mitochondrial temperature increase in BA treated with 10 μmol/L CCCP is ~0.057 K/s at 306.15 K

To verify the theoretical calculation, we have estimated the maximum transient rate of mitochondrial temperature increase in BA induced by 10 μmol/L CCCP, which is indeed on the order of ~10^−2^–10^−1^ K/s (Fig. 4). Clearly, Baffou’s model is too simple to give accurate estimations since they have wrongly used the steady state without temporal factor. In addition, they have also ignored that there are various internal heat sources in cell (Ye *et al*. 2011; Kiyonaka *et al*. 2013; Kucsko *et al*. 2013; Arai *et al*. 2014, 2015; Homma *et al*. 2015), and that cell is nonhomogeneous and rich in membrane structures where lipid bilayer has a low thermal conductivity (0.25 W/(mK)) (Nakano *et al*. 2010).

In summary, we have practically demonstrated uneven mitochondrial thermal maps in living cells, theoretically inferred detectable heat sources (mitochondria), and also pointed out the error of Baffou’s critique. RhB-ME-based mito-thermometry makes it easier for large-scale data acquisition, especially for primary cultured cells, such as BA. Our current observations raise open questions about diversely thermogenic responses of individual BA evoked by 0.1 μmol/L NE, for instance, what is the *in vivo* regulatory mechanism to increase the efficacy of NE-induced thermogenesis in BA?

## Methods

### RhB-ME synthesis

A mixture of RhB (500 mg, 1.04 mmol) and thionyl chloride (2 ml) in chloroform (20 ml) was heated to 60 °C and stirred for 10 min. After cooling to room temperature, the mixture was quenched with methanol. The solvent was removed under reduced pressure and purified by prep-HPLC to give compound 375 mg, yield 73%. ^1^H-NMR (600 MHz, CDCl_3_) *δ* 8.30 (d, *J* = 7.2 Hz, 1H), 8.23 (brs, 2H), 7.79–7.82 (m, 1H), 7.73–7.76 (m, 1H), 7.31 (d, *J* = 7.2 Hz, 1H), 7.06 (d, *J* = 9.6 Hz, 2H), 6.82–6.83 (m, 4H), 3.68 (s, 3H), 3.60 (q, *J* = 7.2 Hz, 8H), 1.32 (t, *J* = 7.2 Hz, 12H); ESI-HRMS exact mass calcd for [M]^+^ requires *m*/*z* 457.2486; found *m*/*z* 457.2484.

### The change rate of normalized intensity ratio (Rh800 to RhB-ME) at a temperature

According to Arrhenius Eq. 2, the local/pixel (*j*) fluorescent intensity ratio *r*(*j*) of Rh800 to RhB-ME at temperature *T*(*j*) can be fitted with:

2 $$r\left( j \right) = A{\text{e}}^{{ - \frac{{E_{\text{a}} }}{{k_{\text{B}} T\left( j \right)}}}} ,$$

where *k*_B_ is the Boltzmann constant; *E*_a_ is the observed activation energy, which is experimentally estimated (*E*_a_ = 6.55 kcal/mol, Fig. 4); *A* is a parameter related to imaging setup and experimental settings.

The parameter *A* in Eq. 2 can be canceled out by dividing the ratio value *r*_ref_ of a reference with a measurable temperature *T*_ref_. Thus, the normalized ratio *nr* is a function of *T* and determined by Eq. 3:

3 $${{nr}} = \frac{r}{{r_{\text{ref}} }} = {\text{e}}^{{ - \frac{{E_{\text{a}} \left( {\frac{1}{T} - \frac{1}{{T_{\text{ref}} }}} \right)}}{{k_{\text{B}} }}}}.$$

The change rate of *nr* at a reference temperature *T*_ref_ can be deduced from the derivative of Eq. 3 with respect to *T*, and determined by Eq. 4:

4 $$\it \frac{{\Delta {{nr}}}}{\Delta T} = \frac{{E_{\text{a}} }}{{k_{\text{B}} }}\frac{1}{{T_{\text{ref}}^{2} }}.$$

Full Methods and any associated references are available in the online version of the paper.

## Electronic Supplementary Material

Below is the link to the electronic supplementary material.

Supplementary material 1 (PDF 627 kb)

Supplementary material 2 (PDF 85 kb)

Supplementary material 3 (DOCX 20 kb)

Supplementary material 4 (MOV 2142 kb)

Supplementary material 5 (MOV 2739 kb)

Supplementary material 6 (MOV 2237 kb)
