# Supplementary material for: Dye-based mito-thermometry and its application in thermogenesis of brown adipocytes
Source: Biophys Rep. 2017 May 13;3(4):85–91. doi: 10.1007/s41048-017-0039-6 (PMC5719795; doi:10.1007/s41048-017-0039-6)
Supplement: Supplementary file 1 — Supplementary material 1 (PDF 627 kb) [file 41048_2017_39_MOESM1_ESM.pdf]

Supplemental figures

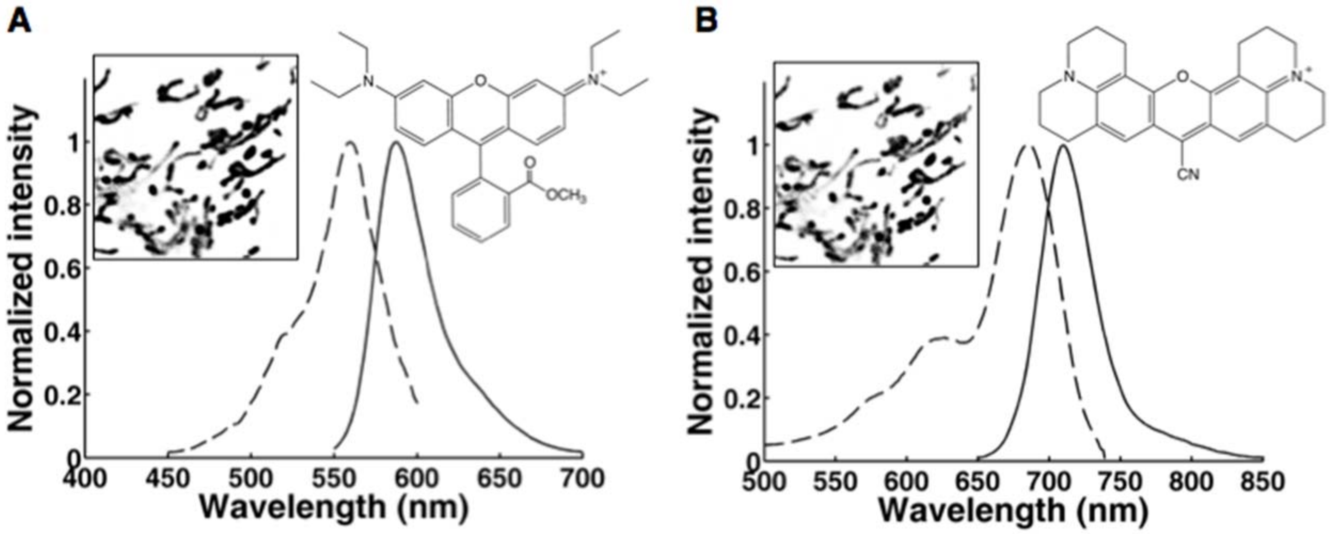

Fig. S1 The spectra and structures of RhB-ME and Rh800. **A** Excitation (*dashed line*) and emission (*solid line*) spectra of RhB-ME. The insets show mitochondria labeled with RhB-ME in HeLa cells and the structure of the RhB-ME. **B** Excitation (*dashed line*) and emission (*solid line*) spectra of Rh800. The insets show mitochondria labeled with Rh800 in HeLa cells and the structure of Rh800

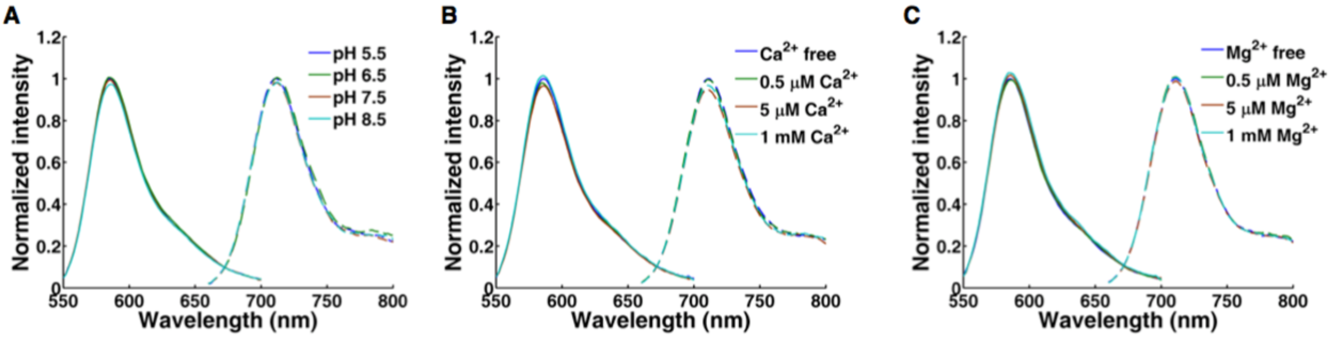

Fig. S2 RhB-ME and Rh800 are insensitive to pH, Ca^{2+} and Mg^{2+}. **A** Emission spectra of 10 μmol/L RhB-ME (*solid lines*) and 10 μmol/L Rh800 (*dashed lines*) at pH 5.5, pH 6.5, pH 7.5 and pH 8.5 respectively. **B** and **C**, Emission spectra of 10 μmol/L RhB-ME (*solid lines*) and 10 μmol/L Rh800 (*dashed lines*) at 0, 0.5, 5, 1000 μmol/L Ca^{2+} (**B**) or Mg^{2+} (**C**) respectively

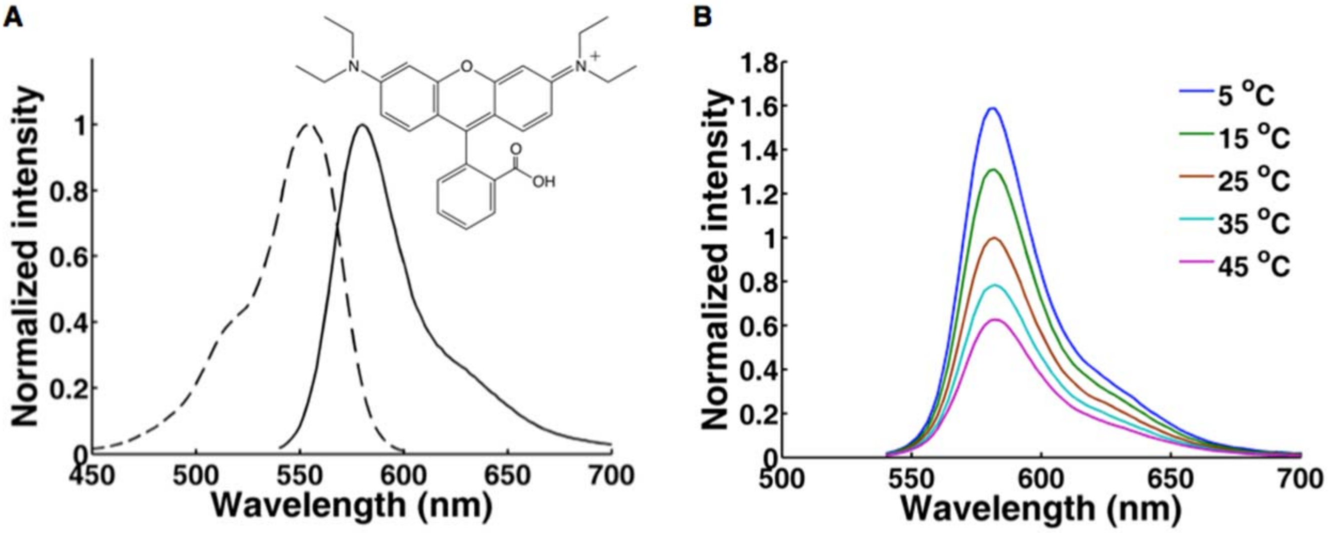

Fig. S3 The spectra and structure of RhB. **A** Excitation (*dashed line*) and emission spectra (*solid lines*) of RhB. The inset shows the structure of the RhB. **B** Emission spectra of RhB from 5 to 45 ℃

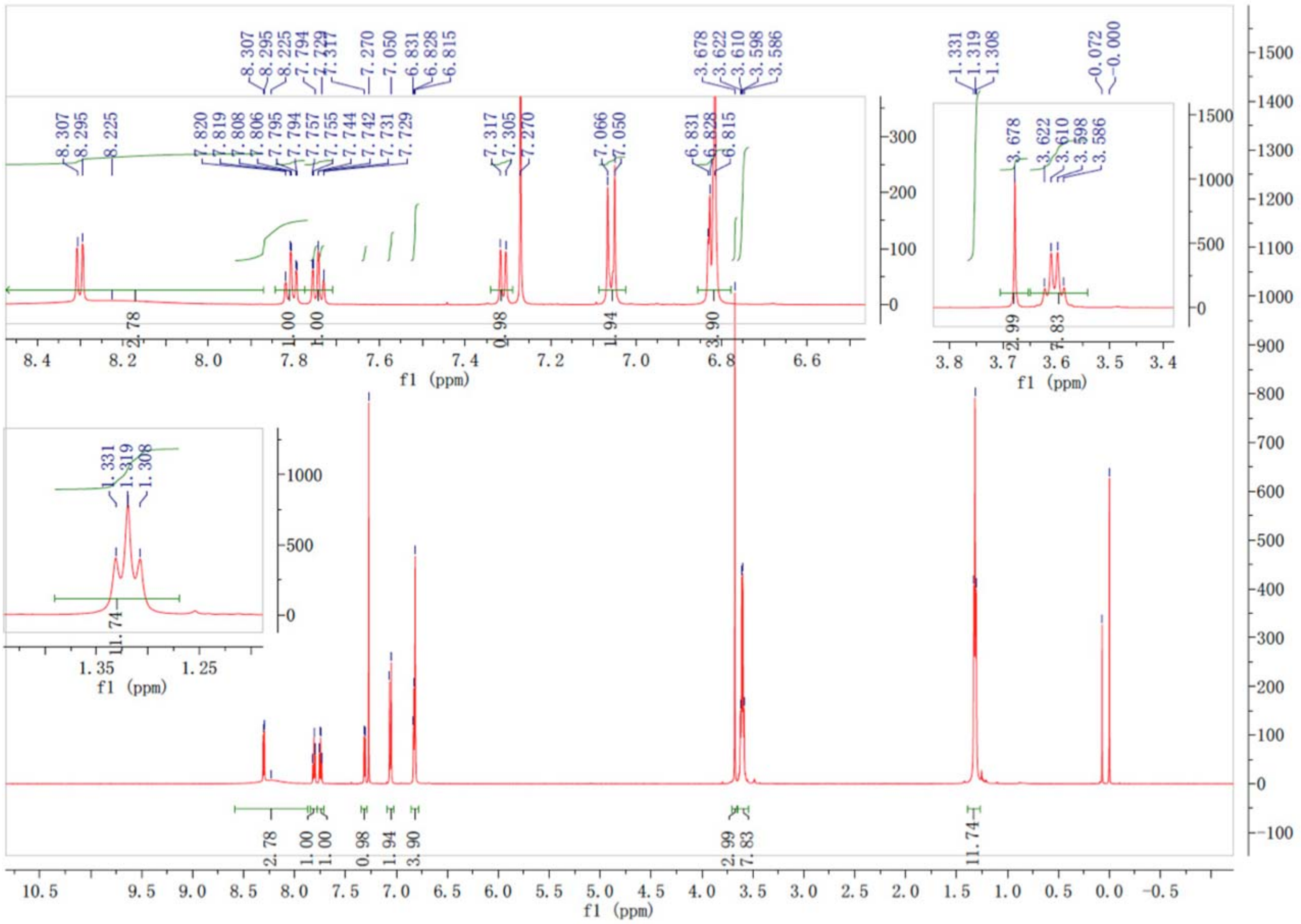

Fig. S4 The ^{1}H-NMR spectral data of RhB-ME at RT. ^{1}H-NMR (600 MHz, $CDCl_3$) δ 8.30 (d, J = 7.2 Hz, 1H), 8.23 (brs, 2H), 7.79–7.82 (m, 1H), 7.73–7.76 (m, 1H), 7.31 (d, J = 7.2 Hz, 1H), 7.06 (d, J = 9.6 Hz, 2H), 6.82–6.83 (m, 4H), 3.68 (s, 3H), 3.60 (q, J = 7.2 Hz, 8H), 1.32 (t, J = 7.2 Hz, 12H)

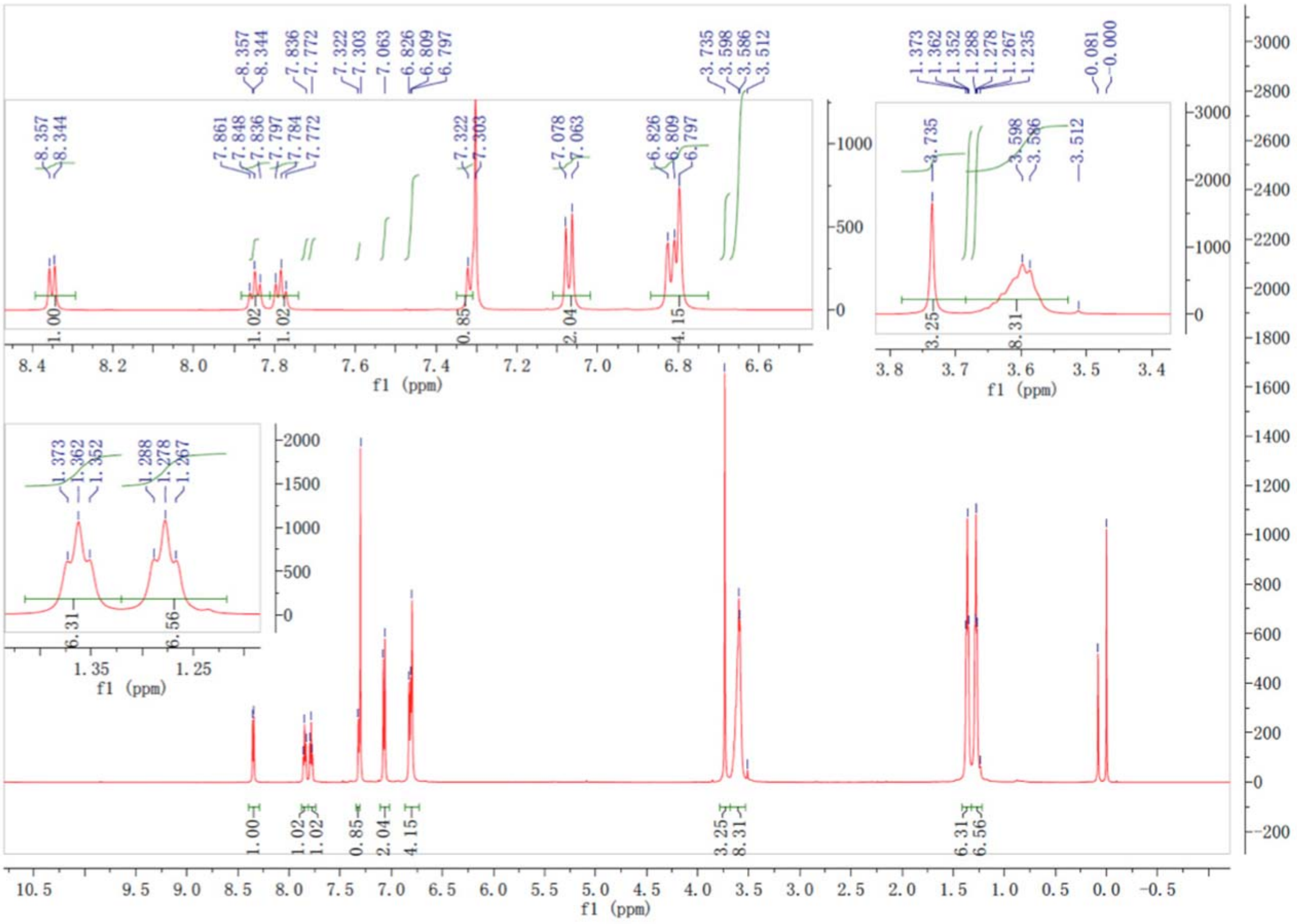

Fig. S5 The ^{1}H-NMR spectral data of RhB-ME at −40 ℃. ^{1}H-NMR (600 MHz, $CDCl_3$) δ 8.35 (d, J = 7.8 Hz, 1H), 7.84–7.86 (m, 1H), 7.77–7.80 (m, 1H), 7.30–7.32 (m, 1H), 7.07 (d, J = 9.0 Hz, 2H), 6.80–6.83 (m, 4H), 3.74 (s, 3H), 3.60 (q, J = 7.2 Hz, 8H), 1.36 (t, J = 6.6 Hz, 6H), 1.28 (t, J = 6.6 Hz, 6H)

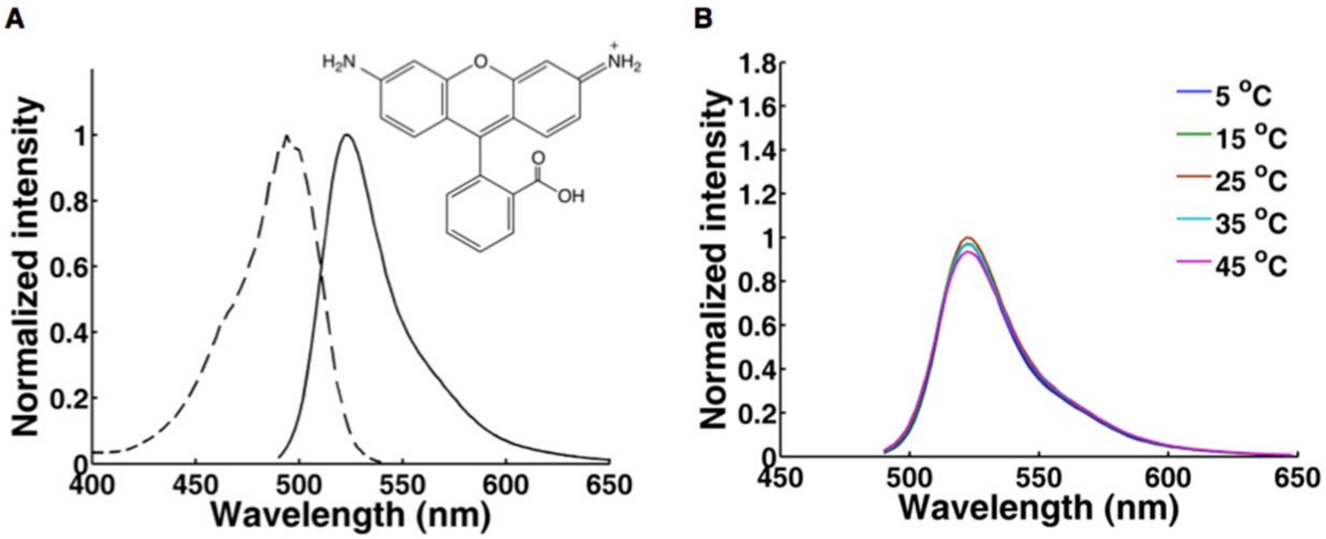

Fig. S6 The spectra and structure of Rh110. **A** Excitation (*dashed line*) and emission spectra (*solid lines*) of Rh110. The inset shows the structure of the Rh110. **B** Emission spectra of Rh110 from 5 to 45 ℃
