# Supplementary material for: Dye-based mito-thermometry and its application in thermogenesis of brown adipocytes
Source: Biophys Rep. 2017 May 13;3(4):85–91. doi: 10.1007/s41048-017-0039-6 (PMC5719795; doi:10.1007/s41048-017-0039-6)
Supplement: Supplementary file 2 — Supplementary material 2 (PDF 85 kb) [file 41048_2017_39_MOESM2_ESM.pdf]

Supplementary Methods

General materials and methods

Rhodamine B (RhB), Rhodamine 800 (Rh800), and carbonyl cyanide m-chlorophenyl hydrazone (CCCP), Collagenase type II and cytosine arabinoside (Ara-C) were purchased from Sigma-Aldrich Corporation (USA). Norepinephrine (NE) was purchased from Santa Cruz Biotechnology, Inc. (USA).

Dulbecco's Modified Eagle Medium (DMEM), fetal bovine serum (FBS), newborn calf serum (NCS), Phosphate-Buffered Salines, pH7.4 (PBS) and Penicillin Streptomycin (Pen Strep)，Coverslips were purchased from Thermo Fisher Scientific Co., Ltd. (USA). Matrigel was purchased from BD Biosciences Company (USA).

C57BL/6J mice were purchased from Sino-British SIPPR/B&K Lab Animal Ltd., Shanghai (China). All experimental procedures and protocols were approved by the Institutional Animal Care and Use Committee of the Institute for Nutritional Sciences, Shanghai Institutes for Biological Sciences, Chinese Academy of Science.

All ^{1}H-NMR spectra (Supplementary Fig. S4 and S5) were obtained using an Agilent 600 MHz DD2 spectrometer. Electrospray ionization (ESI) high-resolution mass spectrometry (HRMS) analysis was carried out on the Agilent 6224 Accurate-Mass TOF LC/MS System. All imaging was performed using confocal microscope FW1000 (Olympus Corporation, Japan).

All data are expressed as mean ± S.D. in text. All data points in figures represent mean ± S.E.M. We accumulated the data for each condition from at least three independent experiments. No statistical methods were used to predetermine sample size.

Spectral scan

Excitation and emission profiles of each dye (10 μmol/L in aqueous solution) were acquired using a temperature-controlled spectrofluorometer (Varian Cary Eclipse, Agilent Technologies, USA) in temperature dependence spectra scanning, and using Varioskan™ Flash Multimode Reader (Thermo Fisher Scientific Co., Ltd., USA) in ions dependences spectra scanning. Excitation profile of RhB-ME was scanned from 450 nm to 600 nm (emission wavelength at 620 nm). Emission profiles of RhB-ME were scanned from 550 nm to 700 nm (excitation wavelength at 530 nm). Excitation profile of Rh800 was scanned from 500 nm to 740 nm (emission wavelength at 760 nm). Emission profiles of Rh800 were scanned from 650 nm to 800 nm (excitation wavelength at 630 nm,). All spectra were normalized to peak values at 25 °C.

Confocal imaging of RhB-ME and Rh800 mixture for temperature curve

A mixture of 10 μmol/L RhB-ME and 10 μmol/L Rh800 in aqueous solution was imaging using confocal microscope with a 20×/0.75 objective (Olympus). The mixture was mildly heated from 18.9 °C to 62.9 °C using Nickel-chromium (NiCr) wire when a time-lapse imaging (interval of 10 s) was performed. A platinum resistance temperature detector Pt100A with a SBWZ PT100 temperature transmitter was employed to monitor the mixture temperature, and a corner of the temperature detector was focused in the imaging field. Two channels of ratiometric imaging were excited with 559 nm and 635 nm lasers, and the fluorescence was collected at 575-620 nm and 655-755 nm simultaneously with largest pinhole setting and the shortest scanning time. Digital signals for imaging events and temperature transmitter signals were recorded with Axon MiniDigi digitizer (Molecular Devices, USA). The time-lapse images were collected at 512 × 512 pixels resolution (12 bit).

Isolation and primary culture of brown adipocytes

Brown adipocytes (BA) were isolated from 3-4weeks male C57BL/6J mice, with a procedure similar to that described by Lucero *et al*. (Lucero and Pappone, 1989). Briefly, mice were kept at 4 °C for overnight with free access to food and water to deplete stored lipid in brown adipose tissues (BAT). The mice were then sacrificed by cervical dislocation and swabbed with 75% ethanol. Interscapular BAT was isolated and placed in isolation buffer (DMEM with 4% NCS). The tissue was minced and digested with 0.2% collagenase type II in a shaking water bath at 37 °C for 30 min (Fain *et al*., 1967). After digesting, discarded the reaction mixture, and the tissue was washed with isolation buffer. Dissociated cells by gently triturating with fire-polish pipettes and washed by centrifugation in PBS. After the final washing, the cells were plated onto 12 mm coverslips (~3×10^4 cells per coverslip). Coverslips were precoated with matrigel. After 2 hours, 2 ml plating medium (DMEM supplemented with 5% FBS, 100 units/mL penicillin and 100 µg/mL streptomycin) was added to each 35 mm dish. From the second day in culture, half of the medium was replaced with feeding medium (plating medium supplemented with Ara-C to inhibit fibroblast proliferation, 2 µmol/L final concentration of Ara-C) every 2 days. Cells were maintained at 37 °C in a humidified atmosphere of 95% air and 5% CO_2, and used for imaging at 3~8 days *in vitro*.

Imaging and cell staining

All imaging was performed using confocal microscope with a 40×/0.95 objective (Olympus) for time-lapse imaging and a 100×/1.4O objective (Olympus) for high-resolution imaging. Cells were co-stained with RhB-ME and Rh800 (20 nmol/L dyes for time-lapse imaging, 50 nmol/L dyes for high resolution imaging) in Tyrode’s solution (in mmol/L: 10 Hepes, 10 glucose, 3 KCl, 145 NaCl, 1.2 $CaCl_2$,

1.2 $MgCl_2$, pH 7.4) for 1 h at 33 °C. The pseudo color of RhB-ME channel is red (excited at 559 nm and collected at 575–620 nm), and Rh800 channel is green (excited at 635 nm and collected at 655–755 nm). All images were collected at 512 × 512 (for time-lapse imaging) and 1600 ×1600 (for high resolution imaging) pixels resolution (12 bit).

Data acquisition and analysis for thermogenesis study

To minimize the heat influence by perfusion solution, time-lapse imaging of BA was performed in 2 mL Tyrode solution rather than in perfusion system. The temperature of experiments was maintained at 33 °C to avoid the probable hyperpyrexia induced by drug treatments. To minimize bleaching and damage to live BA in time-lapse imaging, the lowest intensity of lasers with largest pinhole setting and the shortest scanning time were used, and 101 frames were recorded in a time-lapse imaging at 30 seconds interval. NE (0.1 μmol/L), CCCP (10 μmol/L), or vehicle was injected as soon as the 11th frame of the time-lapse imaging for thermogenesis studies in BA.

After background being removed, ratiometric values of Rh800 channel to RhB-ME channel (simultaneously excited by 635 nm and 559 nm lasers) were calculated pixel by pixel to represent the thermal response of the sample. For noise reduction, the pixels with signal-to-noise ratios less than 1.5 were excluded and 5×5 moving average was used before ratiometric process. According the three-sigma rule, the outlier (99.7% tolerance interval) of the ratios was also excluded. The ratio of each cell was the average ratio of all pixels representing the cell. Since we focus on the thermal responses, the mean ratio of steady state before drug treatments was used to normalize every data point for each cell. All data analysis was performed with MATLAB (MathWorks Inc. USA) and ImageJ (NIH, USA).

References

Fain JN, Reed N and Saperstein R (1967) The isolation and metabolism of brown fat cells. J Biol Chem, 242: 1887–1894

Lucero MT and Pappone PA (1989) Voltage-gated potassium channels in brown fat cells. J Gen Physiol, 93: 451–472
