# Supplementary material for: Dye-based mito-thermometry and its application in thermogenesis of brown adipocytes
Source: Biophys Rep. 2017 May 13;3(4):85–91. doi: 10.1007/s41048-017-0039-6 (PMC5719795; doi:10.1007/s41048-017-0039-6)
Supplement: Supplementary file 3 — Supplementary material 3 (DOCX 20 kb) [file 41048_2017_39_MOESM3_ESM.docx]

**Supplemental video legends**

**Video S1** Subpopulations of NE-induced thermogenesis in BA. The ratiometric pseudocolor movie of 0.1 μmol/L NE-induced thermogenic responses in BA. Scale bar, 20 μm

**Video S2** Subpopulations of NE-induced thermogenesis in BA. The raw channel data of **Video S1** show BA subpopulations after NE treatment. *Red* represents the channel of RhB-ME, while *green* represents Rh800. Scale bar, 20 μm

**Video S3** CCCP-induced thermogenesis in BA. The ratiometric pseudocolor movie of 10 μmol/L CCCP-induced thermogenic responses in BA. Scale bar, 20 μm
